# Simulation of apically grounded cochlear implant stimuli using neural stimulation models

**DOI:** 10.1007/s11548-026-03702-3

**Published:** 2026-05-17

**Authors:** Jared A. Rybarczyk, Erin L. Bratu, Robert F. Labadie, Jack H. Noble

**Affiliations:** 1 Vanderbilt University, Nashville, USA; 2 Medical University of South Carolina, Charleston, USA

**Keywords:** Cochlear implants, Image-based modeling, Neural stimulation, Auditory nerve fiber modeling

## Abstract

**Purpose:**

Cochlear implant (CI) electrode arrays typically do not extend along the entire cochlear duct, often resulting in poor stimulation of the most apical nerves. To better stimulate apical auditory nerve fibers (ANFs), previous studies have examined placing an isolated ground contact within the helicotrema, providing the ability to direct current apically. In this study, we utilize finite difference method (FDM) simulations and computational ANF models to study the effect of apical ground placement on apical ANF stimulation.

**Methods:**

High-resolution resistivity maps based on postoperative CT scans for 3 CI patients were used in FDM-based generation of cochlear voltage maps. Models corresponding to the use of a typical extracochlear ground and a helicotrema ground were used to drive ANF models, allowing for generation of activation thresholds at each of 75 modeled ANF bundles throughout the cochlea. Thresholds between the two models were compared to assess the effects of an apical grounding contact on improving apical ANF stimulation.

**Results:**

In all 3 patients, we observe a relative reduction in stimulation thresholds for apical ANFs when an apical ground is present as opposed to an extracochlear ground. Additionally, when an apical ground is used, we observe an increase in stimulation thresholds for more basal ANFs.

**Conclusions:**

Apical ground contacts can increase activation of typically under-stimulated low frequency ANFs without the need for longer contact arrays, providing a solution for improving low frequency sound perception and potentially leading to better hearing quality.

## Introduction

Sensorineural hearing loss affects an estimated 430 million people worldwide [1]. For patients with moderate to profound hearing loss, the standard of care is the cochlear implant (CI). While there are many causes for sensorineural hearing loss, most cases stem from sensory hearing loss, involving defects and degeneration of hair cells [2, 3]. CIs treat hearing loss by transducing sound into electrical signals. These raw electrical signals are transformed into pulse trains that are then delivered to 12—22 intracochlear electrode array contacts, directly stimulating the auditory nerve fiber (ANF) cell bodies present in the spiral ganglion of the cochlear modiolus. Among patients with successful atraumatic implantation, average word perception ability improved by 45%, with 82% of adults with postlingual hearing loss and 53% of adults with prelingual hearing loss improving their speech perception ability by > 15% [4].

Successful CI-based hearing restoration is dependent on the electro-neural interface. Though there are many arrays offered by multiple manufacturers, most arrays conform to one of two types: lateral wall (LW) or modiolar hugging (MH) [5]. Both LW and MH arrays are ideally inserted into the scala tympani (ST), with the differences between them coming from their proximity to the cochlear spiral ganglion. Straight LW arrays sit along the lateral wall of the ST while MH arrays are pre-curved, sitting directly against the modiolus. The potential benefit of MH arrays is the reduced proximity from the electrode to the ANF, resulting in lower power requirements to achieve the same effect as LW arrays and better overall ANF stimulation due to reduced current spread. However, MH arrays have a higher incidence of scalar translocation compared to LW arrays, potentially damaging the basilar membrane separating the ST and the scala vestibuli (SV) and resulting in worsened speech recognition outcomes [6, 7].

The overall insertion depth of the array plays a large role in restorative ability and is heavily dependent on individual patient anatomy. In most cases, electrode arrays can be safely inserted approximately 400–450 degrees or 1.11–1.25 turns into the cochlea. This depth of insertion allows for good coverage of the more basal ANFs which encode higher frequency sounds and can produce acceptable hearing outcomes for patients who have residual low frequency perception. However, spiral ganglion cells are present throughout 1.75–1.85 turns of the cochlea, equivalent to an insertion depth of 630–660 degrees [8, 9]. For patients with profound deafness who lack both high and low frequency auditory perception, the standard electrode array insertion depth neglects a significant portion of lower frequency encoding ANFs, resulting in hearing that is shifted toward higher pitches and worsened patient outcomes in speech recognition in noisy conditions [10]. The most straightforward approach to address this issue is simply increasing the length of the electrode array, which increases the risk for intracochlear trauma and scalar translocation. Additionally, in the case of MH arrays, the technology to maintain curvature while allowing for deeper insertion beyond 1.25 turns is still under development.

An alternative approach that has received recent interest is the use of an apically placed grounding contact. In a typical CI, current flows from the stimulating electrode(s) to an extracochlear ground, often at the CI case, which is implanted under the temporalis muscle. This allows current to flow out of the cochlea primarily through the internal auditory canal and the round and oval windows. Recently, another group has proposed placing a grounding contact within the cochlear helicotrema via an apical cochleostomy. The usage of this apical ground theoretically allows for current from the most apical stimulating electrodes to travel deeper into the cochlea, beyond what typical stimulation modes would typically reach, and provide some level of stimulation to the otherwise neglected low frequency encoding ANFs. Two main studies from Landsberger et al. [11, 12] have examined both the feasibility and impact of apical ground contacts. The first study examined qualitative pitch scaling and speech perception outcomes on 3 patients who underwent successful apical cochleostomies. The results of this study showed that placement of an apical ground contact was clinically feasible, and that its usage caused lowered pitch perception and improved speech recognition abilities relative to monopolar stimulation with a case ground. The second study aimed to examine the degree of current steering provided by an apical ground via electrical voltage tomography (EVT) measurements on 2 cadaveric cochleae implanted with apical ground contacts. The results of this study supported the hypothesis that an apical ground increased current flow toward the apex when the contact was placed in perilymph.

Both studies highlight the potential usage of apical ground contacts to overcome the issues faced by the average length electrode array in stimulating apical ANFs. One key area that could provide an enhanced insight into the biophysical effects of an apical ground contact on the electro-anatomical environment is the usage of computational nerve models. Due to the nature of these previous apical ground studies, only qualitative speech outcomes and electric field effects could be directly studied, with no examination of ANF behavior. In this study, we aim to bridge the gap and provide a better understanding of the direct effects of an apical ground on ANF stimulation. We accomplish this by using digital twins, computationally modeling the cochlear environment and using finite difference method (FDM) simulations to drive nerve models that provide enhanced information on ANF stimulation thresholds that can help explain hearing outcomes.

Previous simulation studies in cochlear implantation have commonly utilized FDM, the finite element method (FEM), or the boundary element method (BEM), with FEM being the standard method for its ability to handle complex cochlear geometries and computational efficiency [13–18]. At an adequate grid resolution, FDM allows us to create accurate patient-specific models with realistic tissue types, similar to other methods. FDM’s uniform discretization does not require refinement of tissue mesh quality, making it more robust to numerical errors and solution instability at areas of interest than FEM or BEM. As such, FDM can obtain comparable solution quality to more complex methods without refinement at the cost of computational overhead, making it an appropriate method for this study [19].

## Material and methods

This study relies on retrospective analysis of a cohort of 3 single-sided cochlear implant users (2 MH arrays, 1 LW array, referred to as MH1, MH2, and LW1). For reference, patient anatomical measurements including elliptical A (major axis) and B (minor axis) widths as well as basolateral turn (BTL) and total cochlear duct lengths (CDL) are shown in Table 1. To obtain realistic simulations of neural fiber activity, our methodology can be split into two parts: patient-specific FDM-based electro-anatomical cochlear modeling and biophysical ANF modeling. Specific implementation details can be found in the appendix. All electro-anatomical modeling and graphing were performed in MATLAB R2025a, and all biophysical ANF models were implemented in Python 3.11 [20, 21]. Additionally, our biophysical ANF models utilize the NEURON 8.7.1 scripting language and associated Python package [22].

**Table 1:** Patient cochlear anatomical measurements. BTL and CDL were estimated using the A and B lengths via a modified version of Escudé’s formula [23, 24]. All lengths shown are reported in millimeters

Patient	A	B	BTL	CDL
MH1	8.9	6.5	21.5	40.7
MH2	9.8	7.1	23.6	44.6
LW1	8.9	6.4	21.3	40.3

### Electro-anatomical cochlear modeling

For each patient, we create two different types of electro-anatomical models: one that models the use of the standard case ground and one that models the apical ground. Using previously acquired post-implantation CT scans, we construct resistivity maps of patient tissues corresponding to 4 different tissue classes (ST/SV, Modiolus, Soft Tissue, and Bone). For each patient, we hold resistivity values across tissue type constant, assigning values based on previous results [25]. We then approximate the positions of 75 evenly spaced ANF bundles (spanning spiral ganglion cell bodies to peripheral terminals) within the cochlea and use these resistivity maps as the solution domain for our FDM simulations. Each ANF bundle represents approximately 500 fibers.

**Fig. 1:**
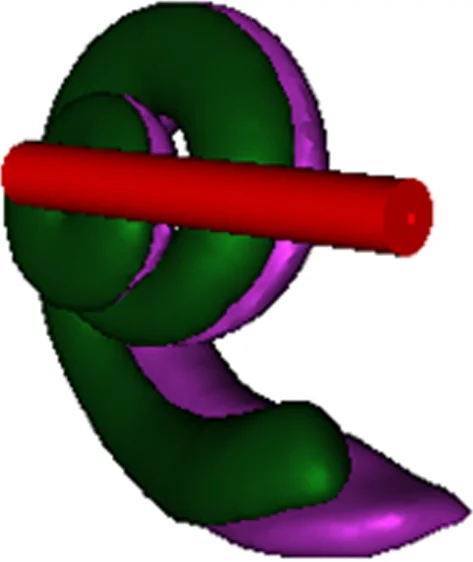
Example placement of the apical ground in patient MH2’s helicotrema, showing the ST (purple), SV (green), and ground lead (red)

For the model that utilizes the standard case ground, we define ground conditions to be at the edge of the solution domain in order to approximate the spread of current due to a comparatively distant ground contact. For the model that utilizes the apical ground, we model an approximately 0.75 mm thick and 5 mm long cylindrical contact that has a trajectory aligned with an insertion path through the facial recess terminating in the helicotrema. The importance of the apical ground being placed in the fluid as opposed to in tissue is discussed in previous work [11]. An example placement is shown in Fig. 1. The main concern of a uniform grid-based volume conduction model such as FDM is its ability to capture complex geometries. To remedy this, we ensure that our grid discretization is at a fine enough resolution to accurately represent meaningful cochlear details, specifically the boundaries between tissue types.

After approximating a solution via FDM, we create a voltage map that stores the voltage at all voxels within the solution domain. The solution scheme generates one voltage map per electrode, such that voltage map *N* corresponds to the voltage map generated by stimulating electrode *N*.

We sample the resulting voltage maps at up to 85 points along each of our 75 ANF bundles. These 85 points correspond to the 3 main components of our ANF model: the peripheral axon, the soma, and the central “distal” axon. Thus, our final result for the FDM portion of our electro-anatomical model is a set of voltage maps sampled at ANF points which are then used to drive our biophysical models.

### Biophysical ANF modeling

We utilize the Briaire and Frijns auditory nerve compartment model to simulate our ANF bundles [26]. The Briaire and Frijns model is a nonlinear active fiber model representing a bipolar neuron with a high spontaneous fire rate. We have developed an optimization routine to find, for a given fiber, the threshold amplitude for a given stimulus required for the compartment model to predict an action potential would be generated. In our case, the stimulus is derived from the previously generated voltage maps, assigning stimulation to different nodes of the fiber model from the sampled voltages. Thus, for a given electrical stimulation we can derive the activation thresholds for our set of modeled nerve fibers. Specifically, we define our stimulation to be a biphasic monopolar pulse of 25 µs pulse width, similar to standard cochlear implant stimulation. The resulting thresholds describe the level of stimulation required to cause an action potential in at least half of the individual ANFs present in a given bundle. For any given stimulation, we limit our possible fiber activation thresholds to a range of 0–5000 µA. For each patient we examine the differences in both ANF voltage maps and ANF thresholds for both stimulation using the case ground and stimulation using the apical ground. For both the single fiber voltage plots and ANF voltage maps, the first 32–33 segments correspond to the peripheral axon, the next 9–10 segments correspond to the pre-somatic and somatic region, and the remaining 43 segments correspond to the central axon that projects into the modiolus and spiral ganglion.

## Results

### Simulation quality

We begin our presentation of results with a discussion of simulation quality. Because our voltage maps and neural stimulation results are dependent upon an FDM solution with low error, we use the absolute current difference as a measure of simulation quality and continuity. In an arbitrary electrostatic simulation with infinite numerical precision, we would expect the absolute current difference between the source nodes (the electrodes) and the sink nodes (the case or apical ground) to be exactly 0, indicating a solution approximation with perfect continuity. For our FDM simulations, we recorded absolute current differences below $1*{10}^{-7}$ µA, with average absolute current differences on the order of $1*{10}^{-9}$ µA. These results are acceptably close to 0 for the given domain.

### Single fiber voltage profiles

**Fig. 2:**
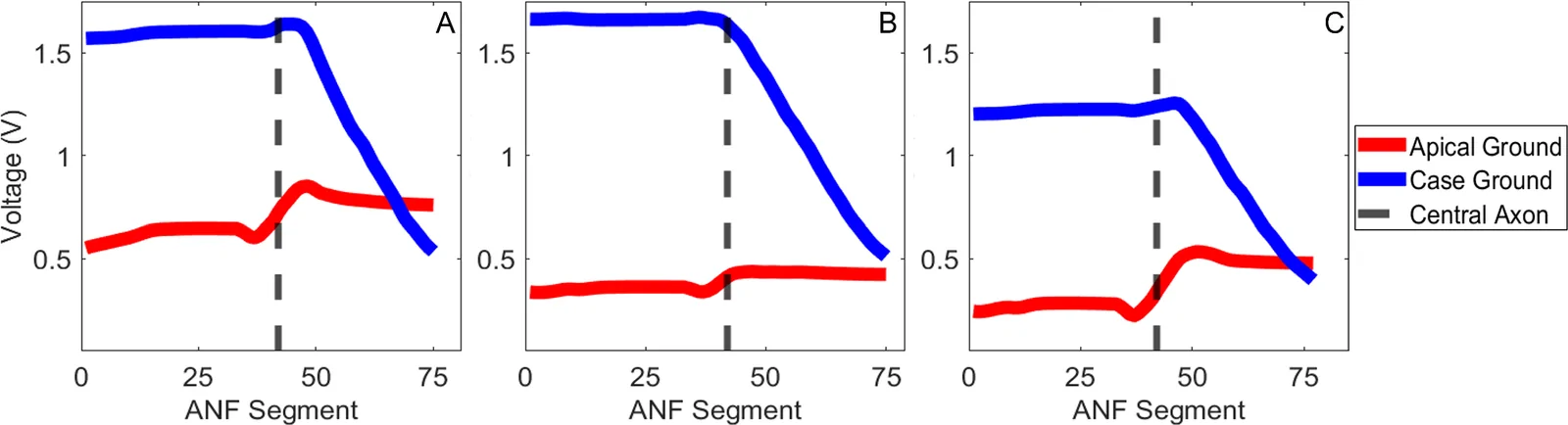
**a**–**c** Patient MH1, LW1, and MH2’s (sub-figures A, B, and C, respectively) voltage plots for apical and case ground simulations at the 75th ANF bundle due to stimulation from the 22nd electrode. The vertical axis represents voltage level along the ANF bundle while the horizontal axis represents segments of the ANF bundle, from most peripheral (1) to most central (75). The red line represents the voltages observed in the apical ground stimulation scheme. The blue line represents voltages observed in the case ground stimulation scheme. The dashed line represents the approximate location of the central axon

Figure 2a-c shows simulated voltage values along the most apical (75th) ANF due to stimulation from the most apical electrode for each of our simulated ground conditions. For all 3 patients, we observe that the most peripheral portion of the ANF bundle receives proportionally higher voltage from the usage of a case ground, while the body of the ANF within the spiral ganglion receives higher voltage from the usage of the apical ground. Specifically, for all 3 patients, the case ground provides a nearly constant level of voltage along the ANF periphery, somatic region, and beginning portions of the central axon before linearly decreasing along the deeper central axonic nodes. Comparatively, in the apical ground simulation, all 3 patients exhibit a roughly constant level of voltage along the peripheral axon before experiencing a slight dip and increase around the body before remaining at constantly elevated levels for the entirety of the central axon. As ANF activation is largely controlled by the 2nd spatial derivative of voltage, we can expect alterations in activity based on the observed differences in voltage along the most apical fiber.

### ANF voltage distributions

**Fig. 3:**
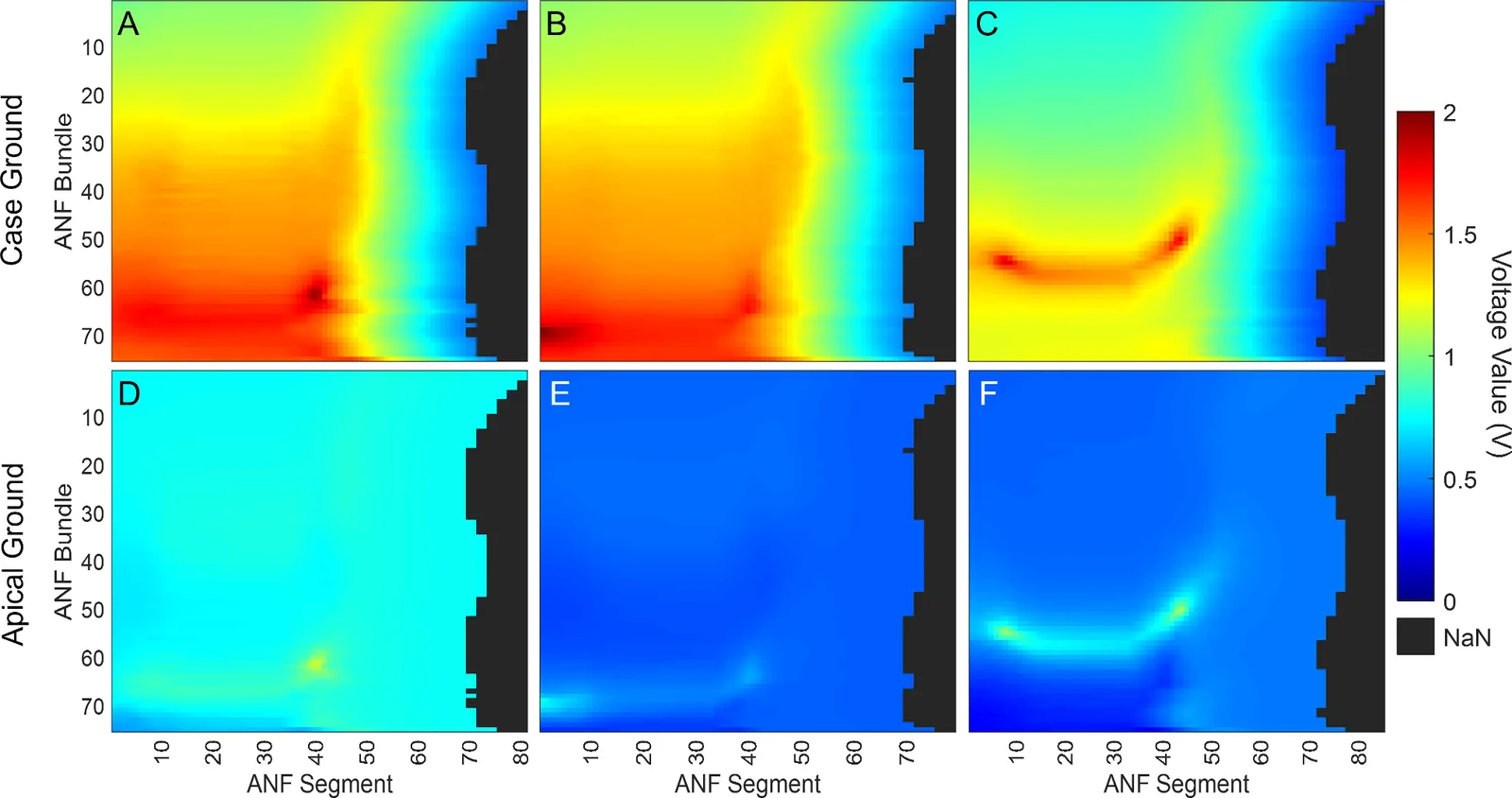
**a**–**f** Patient ANF voltage distributions when the most apical electrode is active. The vertical axis represents each of the 75 ANF bundles (ordered from most basal, 1, to most apical, 75). The horizontal axis represents voxels along those bundles (ordered from most peripheral, 1, to most central, 75–80 depending on patient). Parts a–c show the voltage distribution due to case ground stimulation schemes for patients MH1, LW1, and MH2, respectively, while parts d–f show the voltage distribution due to apical ground stimulation schemes for patients MH1, LW1, and MH2, respectively

Figure 3a–f shows the variations in predicted voltage due to stimulation from the most apical electrode across all 75 ANF bundles, with parts a–c showing the voltage distributions due to case ground schemes and parts d–f showing the distributions for apical ground schemes for patients MH1, LW1, and MH2, respectively. For both apical and case ground maps, we observe peaks in similarly apical areas. However, in the apical ground maps, the voltage remains proportionally higher along all ANFs than in the case ground maps. As such, the gradient in the apical ground voltage maps is steepest around apical ANFs and shallower around the more basal ANFs. Comparatively, the case ground voltage maps show steep gradients around both apical and basal ANFs, as well as a greater overall magnitude of voltage within the cochlea.

### ANF activation thresholds

**Fig. 4:**
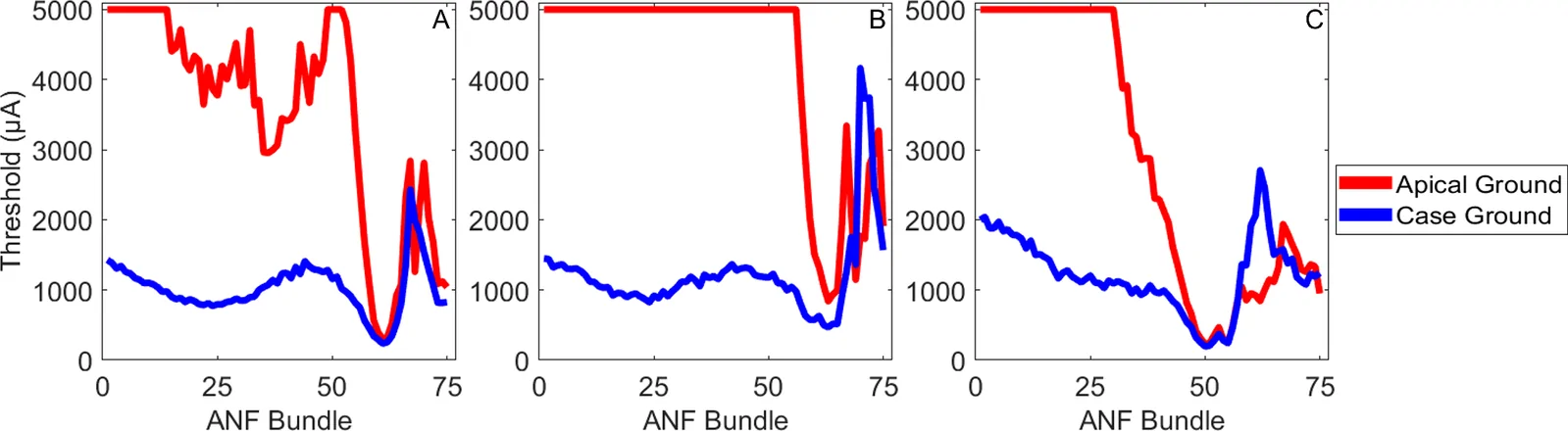
**a**–**c** Patient MH1, LW1, and MH2’s (sub-figures A, B, and C, respectively) ANF activation thresholds due to stimulation from the most apical electrode. The vertical axis represents the activation threshold for a given ANF bundle. The horizontal axis represents each of the 75 ANF bundles throughout the cochlea, ranging from most basal (1) to most apical (75). The red line represents the thresholds observed in the apical ground stimulation scheme. The blue line represents the thresholds observed in the case ground stimulation scheme

Figure 4a-c shows the neural stimulation thresholds across all 75 ANFs for the apical and case ground schemes due to stimulation from the most apical electrode. In the apical ground scheme, across all patients’ results, we observe an increase in activation thresholds for the more basal ANFs as compared to the case ground scheme. Specifically, thresholds in case ground schemes remain relatively flat across the more basal ANFs, experiencing a small dip and subsequent peak at the apical ANFs when exposed to stimulation. Comparatively, in apical ground schemes the thresholds lie beyond our maximum tested current levels of 5A for basal ANFs, experiencing a substantial dip in the same apical cochlear region as case ground schemes with a comparatively minor spike at the most apical end of the cochlea. This dip correlates with the group of ANF bundles that are the easiest to activate via stimulation from the most apical contact in the array.

To further quantify the differences in our threshold results, we introduce a measure of center frequency shift, which scales the proportion of fibers that would be active for a given threshold across all 75 bundles to approximate the center frequency across all fibers. We calculate this measure, *M*, as the weighted average of the sum of the proportions of active fibers, with weightings given by the characteristic frequency of each of the 75 ANF bundles:

1 $$M =\frac{{\sum}_{n=1}^{75}\left({\upomega}_{n}*{\upalpha}_{n}\right)}{{\sum}_{n=1}^{75}\left({\upalpha}_{n}\right)}$$

where α is the proportion of active fibers and ω is the characteristic ANF bundle frequency. For patient MH1, LW1, and MH2 using an apical ground scheme, we observe *M* values of 918, 741, and 2019 Hz, respectively. Conversely, when a case ground scheme is used, we observe *M* values of 1940, 2789, and 2153 Hz. These differences in center frequency correspond to a 1.079, 1.911, and 0.093 octave shift lower for patients MH1, LW1, and MH2, respectively.

## Discussion

In this study, we compared the results of our FDM simulated voltage maps with the EVT results previously presented in [11]. In the prior study, increased current flow toward the most apical portion of the cochlea was observed when a ground contact was placed in the perilymph within the cochlear helicotrema. Our results clearly reflect this, as comparing the voltage map results between the apical ground and case ground scheme show proportionally higher voltage levels at the soma and central axon for apical schemes. While both the apical and case ground voltage maps show peaks in similar areas, the overall altered voltage value suggests that the presence of an apical ground plays a significant role in reshaping current flow in the cochlea.

This theory is further supported by the voltage plots. The voltage plots show a clear shift with respect to voltage levels in each of the simulation cases, with case ground schemes providing proportionally higher voltage levels to peripheral areas of the ANF than central areas. The inverse is true for apical ground schemes where higher voltages were observed at central and somatic portions of the ANF than the periphery. The steep drop-off of voltage along the central ANF portions in case ground voltage plots as compared to the constant level seen in the apical ground plots further supports the idea that the flow of current remains intracochlear in the presence of an apical ground. The possible resulting improved stimulation to spiral ganglion cells has been studied in the context of improving hearing outcomes and neural health, meaning that any increased spiral ganglion stimulation from the use of an apical electrode could have downstream benefits beyond better apical fiber activation [27, 28].

The results in this study provide further insights into the neural and biophysical effects of an apical ground through the usage of computational ANF models. In a previous study, the qualitative effects of an apical ground placed via apical cochleostomy on 3 adult patients were examined [12]. The results of this study found that all 3 patients, in addition to having no loss in speech recognition, experienced perceived lowering of pitch. The results of our neural threshold simulations reflect this. As the activation function of an ANF is controlled largely by the second derivative of the voltage, the shallower gradient of the ANF voltage map along the more basal ANFs seen in the presence of an apical ground could indicate less activation among these fibers. The comparatively steep gradients observed in the case ground simulations would then indicate less specific apical ANF activation. When comparing the threshold results of the case and apical ground-based simulations, we can clearly see that the case ground exhibits higher thresholds toward more apical fibers and lower thresholds toward more basal fibers compared to the apical ground. Though thresholds toward more apical fibers are not necessarily lower than those in case ground schemes, thresholds toward more basal fibers are greatly increased. This increase could potentially result in reduced ability of basal fibers to be stimulated compared to apical fibers, resulting in the lower pitch perception that was previously reported. This lower perceived pitch phenomenon is also supported by our fiber shift measurements, with all patients exhibiting apical shifts of their center frequency with an apical ground.

Future consideration should be given to two main areas. The first should be increasing the number of patients whose data is included in these simulations to improve statistical power and further verify the results observed in this study. The second area of consideration is model accuracy and validity. A concern when working with any computational model is its ability to accurately represent the studied behavior. Previous comparisons between in vivo experimental data and three of the most commonly used ANF cable compartment models (the Rattay, Smith Hakenom, and Briaire Frijns models) determined that none of the three could perfectly represent the experimental findings [29]. For some aspects, such as neuronal temporal dynamics in response to pulse train stimulation, all three models failed to replicate in vivo results due to a lack of representation in temporal adaptation and integration. This disparity highlights the gap between model and reality, further emphasizing the need for extended human ANF characterization. However, for other aspects, such as response to a single pulse stimulation similar to what was implemented in this study, all three models fell into an acceptable range of experimental data points. As such, possible future work could seek to compare the thresholds generated by the other neuronal models in response to apical stimulation in order to determine bounds on realistic behavior. Additionally, our utilized ANF model is based upon healthy fibers. In a realistic CI user, a sizable portion of the fibers in an arbitrary ANF bundle will be degenerated, with decreased excitability. Including degenerated fibers in our analysis would improve the realism and clinical applicability of our results.

## Conclusion

The placement of an apical ground contact has been shown to have measurable effects on both the intracochlear electrical environment and the neural stimulation patterns of ANFs. Particularly, apical grounds increase the flow of current toward the cochlear apex, as well as leading to shallower voltage gradients in more basal regions. With the shallower gradients comes a shift in ANF stimulation thresholds, with apical grounds causing an increase in thresholds for ANFs that are more basal than the stimulating electrode and leading to an apical spatial shift in stimulated ANFs. The results of these simulations both verify and explain results seen in prior research and suggest the usability of apical ground contacts in improving hearing outcomes.

## Appendix

### CT Preprocessing

We obtain both pre- and postoperative CT images of all CI patients at standard resolution. To segment structures-of-interest (SOI), we utilize the methodologies described in [30–33]. Active shape models of SOI based on statistical atlases are used to create patient-specific segmentations from the acquired CT images. Following segmentation, we utilize thin-plate-splines registration on the SOI between patient CT and 9 cadaveric micro-CTs. Using a majority vote scheme between the 9 registrations, we produce a high-resolution tissue class map with a voxel size of 0.072 mm for our patient anatomy. Our final class map defines 4 tissue types, perilymph with resistivity 100Ωcm, soft tissue with resistivity 900Ωcm, neural tissue with resistivity 300Ωcm, and bone with resistivity 9000Ωcm. We assign air an infinite resistivity value. We then automatically define 75 evenly spaced ANF bundles throughout the cochlea using the techniques defined in [34]. Geodesic paths are used in conjunction with 5 cochlear landmarks to assign ANF trajectories along a cylindrical coordinate system that follows the spiral of the cochlea. Following ANF placement, voxels within the ST are overwritten to realistically place the CI electrode array.

### Volume conduction model

The equation of interest for our electrostatics simulation is Poisson’s equation:

2 $${\nabla }^{2}V=\frac{-\uprho }{{\upepsilon}_{0}}$$

where *V* is electrostatic potential, ρ is charge density, and ${\upepsilon}_{0}$ is the permittivity of free space. To solve this equation via FDM, we must approximate the differential Laplacian operator, ${\nabla }^{2}$. Our FDM implementation utilizes the central difference approximation given by the following:

3 $$\frac{{\partial }^{2}u}{\partial {x}^{2}}\approx \frac{{u}_{x+1,y,z}-2{u}_{x,y,z}+{u}_{x-1,y,z}}{{\left(\Delta x\right)}^{2}}$$

where $\frac{{\partial }^{2}u}{\partial {x}^{2}}$ is the second derivative of a function u with respect to x. Similar approximations are used for the y and z direction. For a 3D volume, the solution given by this approximation is reliant on the values of the 6 neighbors of any arbitrary voxel not on the boundary of the domain, corresponding to each of the 6 faces of the central voxel. We can rewrite the central difference approximation to set up a linear system of equations given by:

4 $${A}_{nxn}{x}_{nx1}={b}_{nx1}$$

where *A* is an nxn matrix that defines voxel relationships, *x* is an nx1 vector of unknown voltage values, and *b* is an nx1 vector of known values (corresponding to our sources and sinks). For our 251x251x151 voxel CT volume, n is equivalent to roughly 9.5 million, where at most 7 values per row are non-zero. As such, we can treat *A* as a sparse matrix.

We utilize both Dirichlet and Neumann boundary conditions (BCs). In case ground simulations we assign the boundaries of the domain Dirichlet BCs, forcing our solution to 0 along the edges and approximating the flow of current to a comparatively distant ground contact. In apical ground simulations, we assign Dirichlet BCs to the apical ground. We assign the grid boundaries Neumann BCs, which control the outward normal derivative, keeping it continuous from the voxels’ interior to the boundary. As a result of modeling air at infinite resistivity, we utilize Neumann BCs within the volume at interfaces of tissue and air to enable numerical stability in our solution, as the large difference between tissue resistivity and a realistic finite-valued air resistivity in our solution would cause the *A* matrix to be ill-conditioned. In an ill-conditioned matrix, numerical round-off errors can compound throughout the iterative solution process, resulting in outputs that do not adequately follow the physics of the underlying partial differential equation. By enforcing Neumann BCs at these interfaces, we force the derivative of the solution to behave in such a way that smoothness and continuity are preserved. We utilize the stabilized biconjugate gradient method with LU preconditioning and GPU parallelization to reach a final voltage map across the solution domain for stimulation provided by each of the 22 electrodes.

### ANF model

We utilize the compartment fiber model described in [26]. We model ANFs as 3 main units: peripheral axons, soma, and central axons. These units are subdivided such that our ANF morphology consists of 7 peripheral, 3 somatic, and 30 central segments. Per the Briaire and Frijns biophysical model, to relate extracellular and intracellular potentials, our unmyelinated somatic region and peripheral and central Nodes of Ranvier can be described as active parallel RC circuits, while myelinated internodes are treated as passive resistive components [35]. Additionally, to offset the effect of comparatively large somatic diameters on potential propagation, we include a myelinated pre-somatic region in our ANF morphology.
